# Remote assessment of DMFT and number of implants with intraoral digital photography in an elderly patient population – a comparative study

**DOI:** 10.1371/journal.pone.0268360

**Published:** 2022-05-17

**Authors:** Antonio Ciardo, Sarah K. Sonnenschein, Marlinde M. Simon, Maurice Ruetters, Marcia Spindler, Philipp Ziegler, Ingvi Reccius, Alexander-Nicolaus Spies, Jana Kykal, Eva-Marie Baumann, Susanne Fackler, Christopher Büsch, Ti-Sun Kim

**Affiliations:** 1 Section of Periodontology, Department of Conservative Dentistry, Clinic for Oral, Dental and Maxillofacial Diseases, Heidelberg University Hospital, Heidelberg, Germany; 2 Department of Prosthetic Dentistry, Clinic for Oral, Dental and Maxillofacial Diseases, Heidelberg University Hospital, Heidelberg, Germany; 3 Institute of Medical Biometry, Ruprecht-Karls University of Heidelberg, Heidelberg, Germany, Heidelberg, Germany; University of Bern: Universitat Bern, SWITZERLAND

## Abstract

**Objectives:**

This comparative study aimed to evaluate intraoral digital photography (IODP) as assessment-tool for DMFT and number of implants (IMPL) compared to clinical diagnosis (CLIN) in an elderly population with high restorative status. Secondary research questions were whether an additional evaluation of panoramic radiographs (PAN-X) or raters’ clinical experience influence the agreement.

**Methods:**

Fifty patients (70.98±7.60 years) were enrolled for standardized CLIN and IODP. The clinical reference examiner and ten blinded raters evaluated the photographs without and with a PAN-X regarding DMFT and IMPL. CLIN were used as reference standard and differences to IODP and IODP-PAN-X findings were analysed descriptively. To assess intra-rater agreement, pairwise Gwet’s AC1s of the three diagnostic methods CLIN, IODP and IODP+PAN-X were calculated.

**Results:**

Compared to a DMFT of 22.10±3.75 (CLIN), blinded raters evaluated a DMFT of 21.54±3.40 (IODP) and 22.12±3.45 (IODP+PAN-X). Mean values for “Decayed” were 0.18±0.52 (CLIN), 0.45±0.46 (IODP) and 0.48±0.47 (IODP-PAN-X), while 11.02±5.97 (CLIN), 10.66±5.78 (IODP) and 10.93±5.91 (IODP+PAN-X) were determined for “Missing” and 10.90±5.61 (CLIN), 10.43±4.85 (IODP) and 10.71±5.11 (IODP+PAN-X) for “Filled”. IMPL were 0.78±2.04 (CLIN), 0.58±1.43 (IODP), 0.78±2.04 (IODP+PAN-X). Gwet’s AC1 using the mode of the blinded raters’ assessment of "Decayed", "Missing" and IMPL compared to CLIN ranged from 0.81 to 0.89 (IODP) and 0.87 to 1.00 (IODP+PAN-X), while for "Filled" and DMFT they were 0.29 and 0.36 (IODP) as well as 0.33 and 0.36 (IODP+PAN-X), respectively. Clinical experience did not influence the agreement.

**Conclusions:**

Assessment of “Decayed”, “Missing” and IMPL by IODP showed almost perfect agreement, whereas of “Filled” and DMFT revealed fair to moderate agreement with clinical findings. Additional PAN-X-evaluation increased agreement compared to IODP-diagnostics alone. IODP for the assessment of DMFT and IMPL might be a suitable method in large-scale epidemiological studies, considering high agreement in total values and miscellaneous agreement at patient-level.

## Introduction

Caries in permanent teeth remains the most prevalent condition worldwide and has a serious health and economic burden on individuals and society [1, 2]. In the prevention of caries, an early detection of lesions or caries-risk patients is of great importance [3]. In order to investigate the prevalence of untreated or treated caries in clinical or epidemiological studies, the DMFT index proposed by the WHO has been applied for over 80 years and is still frequently used. The index indicates the number of decayed, missing and filled teeth [4, 5]. Traditionally, this index is carried out as a direct clinical assessment with the advantages of visual-tactile inspection of the tooth and restoration surfaces. However, this approach has some limitations. In large-scale epidemiological studies, the inability to blind examiners or the subjective impact of different examiners can represent a risk of bias. Also, a high level of personnel on site and therefore financial effort must be expected. In the context of increasing multi-center, often multi-national studies, these factors are of growing importance. Data aquired by digital imaging could overcome some of these obstacles [6].

Consequently, the prerequisite for using such data, especially in epidemiological studies, is high standardization and reliability of the collected data compared to clinical data.

Digital methods and imaging are already an integral part of routine patient care and research in dentistry and will gain further importance. Digital imaging for on-site diagnostics and treatment planning as well as for remote data acquisition in large-scale epidemiological studies, will become more relevant as artificial intelligence (AI) advances [7–11]. In addition, dental imaging will increasingly be used in teledentistry [12]. With the purpose of overcoming limited access to dental care due to global rural disadvantages and patients’ immobility as a result of illness or old age, technical approaches are emerging: the assessment of medical or dental history via smartphone is already used to carry out preventive measures, dental diagnostics or monitoring, as well as to identify the specific need for dental treatment [12–17]. However, the validation of such approaches is complicated and further concerns, e.g. about data security, remain an additional hurdle [11].

In dentistry, intraoral digital photography is used for diagnostics, documentation, patient communication and education as well as research. Caries diagnostics by dental photographs showed high validity compared to clinical diagnostics as the reference standard [18–20]. Also tooth decay, dental trauma, tooth wear, plaque scores, esthetic scores around implants and orthodontic procedures have been observed via intraoral digital photography [21–28]. Though, many of these comparative studies have been performed in a healthy or young patient population, often in children and adolescents [18, 19, 21–23, 25, 28].

Therefore, the primary objective of this study was to evaluate to what extent an assessment of the DMFT index, i.e. the recording of “decayed”, “missing” and “filled” teeth and the number of implants by means of digital dental photographs agrees with clinical diagnosis in an elderly patient population with high restorative status. Secondary research questions were to what extent the additional evaluation of panoramic radiographs provide a further benefit and whether the clinical experience of the investigators influence the agreement of the assessment methods.

## Materials and methods

This comparative study was approved by the ethics committee of the Medical Faculty of Heidelberg (# S-630/2019) and is in accordance with the ethical standards of the 1964 Helsinki Declaration and its later amendments or comparable ethical standards. The trial was registered at the US National Institute of Health (ClinicalTrials.gov, # NCT04192188). This study is being reported using the STARD statement for diagnostic accuracy studies and the STROBE statement for cross-sectional studies [29, 30].

### Study participants

The study participants represent a subpopulation of a larger case-control study on the effects of antiresorptive medication in supportive periodontal therapy ("SPTunderART"). In the original study, 100 patients from the Section of Periodontology of the Department of Conservative Dentistry at Heidelberg University Hospital were included. For the present study, the available data of the first 50 participants were analyzed. Written informed consent was obtained from all individual participants included in the study.

### Reference standard: Clinical examination

The clinical examination of the patients for the present study was performed by one clinical examiner (AC) with experience in oral diagnostics in the context of epidemiological studies between December 2019 and March 2020. The examination results served as a reference standard.

First, a professional tooth cleaning was performed on all participants. The subsequent intraoral examination for the recording of the number of teeth, restorations and teeth affected by caries was performed by tactile inspection of the teeth with a probe (EXS3A6, Hu-Friedy Mfg. Co., LLC., Frankfurt am Main, Germany), a mouth mirror (DA036R, Aesculap, B. Braun Melsungen AG, Melsungen, Germany) and air blowing. Intraoral illumination was provided by the operating light of the dental chair (Teneo/LED View Plus, Dentsply Sirona Inc., York, USA). A panoramic radiograph was screened and if the most recent one was older than five years, an updated radiograph was acquired in a standardized manner. Patients were positioned in the Frankfurt horizontal plane, a standard bite block was used and the “P1 program” (radiation time: 13,930 ms, tube current: 16 mA, tube voltage: 69 kV) was followed as specified by the manufacturer (Orthophos SL 3D, Dentsply Sirona Inc., York, USA). The findings were documented manually on a sheet and the DMFT index was recorded digitally afterwards.

### Intraoral digital photography

**Fig 1:**
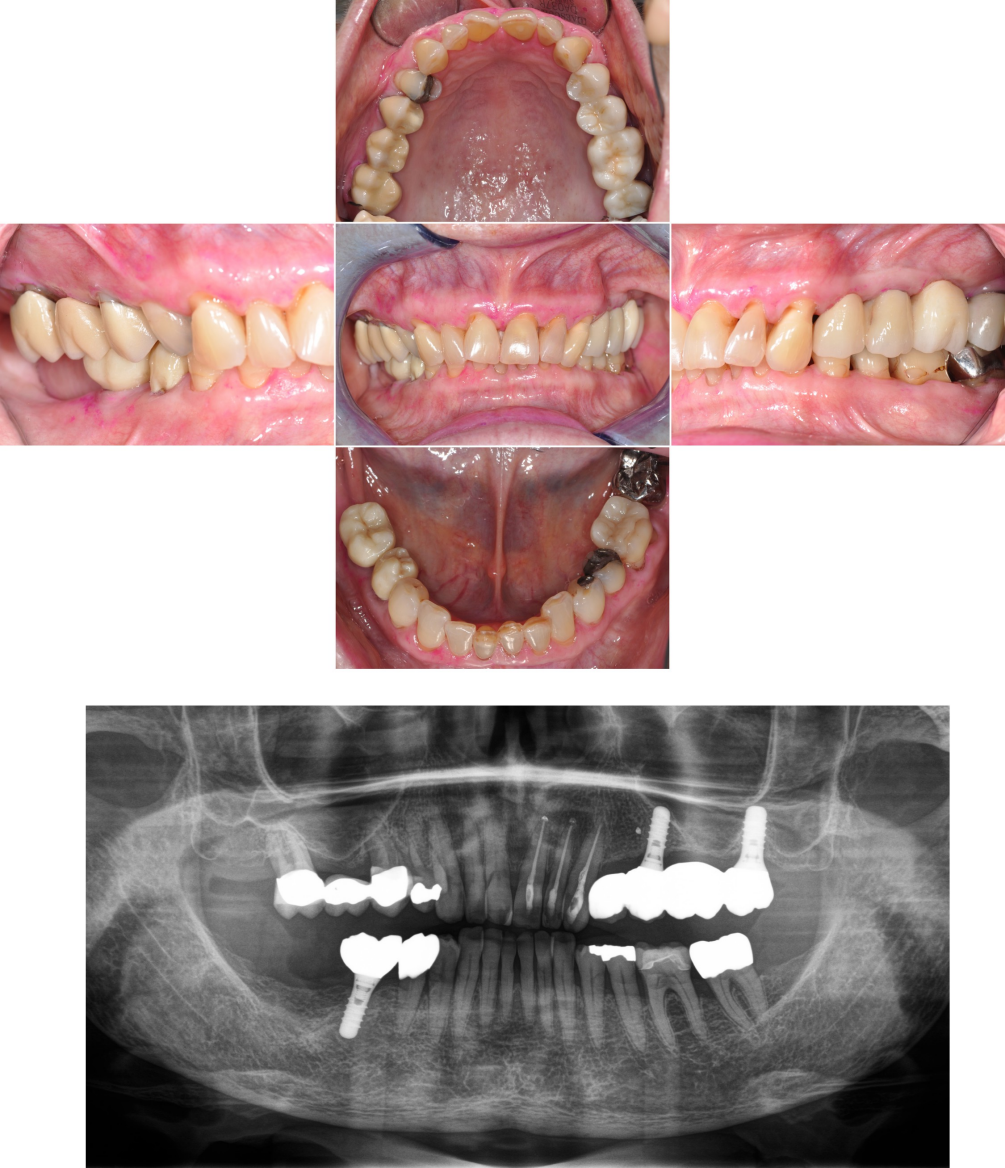
Exemplary intraoral digital photography status (A) and corresponding panoramic radiograph (B).

A complete standardized photographic status comprised of five single photographs was taken of all 50 study participants by the clinical examiner with the help of additional assistance. All participants were positioned on the dental chair in such a way that a perpendicular image of the dentition was possible. All photographs were taken in the same order for every participant. For the first photograph, cheek retractors (Mirahold®, Hager & Werken, Duisburg, Germany) were inserted into the corners of the mouth to allow a frontal photograph to be taken with the dentition in habitual occlusion. The second and third images comprised the buccal representation of the lateral teeth. For this purpose, the cheek retractor was placed into the opposite corner of the mouth and a preheated mouth mirror (“T1”, dps digital-photo-systeme, Enzklösterle, Germany) was inserted into the buccal side to be imaged. The mirror was angulated in a way that the occlusal plane in habitual occlusion was positioned in the center of the image and the lateral teeth could be captured as perpendicular as possible. The fourth image was an occlusal view of the upper jaw. For this purpose, the upper lip was held off by two dental mouth mirrors and a preheated mirror (“XXLH”, dps digital-photo-systeme, Enzklösterle, Germany) was positioned in a way that the occlusal surfaces of all maxillary teeth could be imaged. Analogously, a photograph of the lower jaw was taken as the fifth image. For this purpose, the tongue was moved towards the throat with the mirror. For all intraoral images, the teeth, the gingiva, the oral mucosa of the vestibule and, if necessary, the base of the mouth were cleared of saliva with suction and air blowing. If a removable prosthesis was present, it was additionally photographed extraorally. Fig 1 shows an exemplary intraoral digital photography status and the corresponding panoramic radiograph.

The photographs were taken with a digital single-lens reflex camera (Nikon D90, Nikon Corporation, Minato, Japan) with an 18 to 150 mm lens (AF-S DX Nikkor 18–105 mm f/3.5–5.6G ED VR, Nikon Corporation, Minato, Japan) and a ring flash (EM-140 DG, Sigma Corporation, Kawasaki, Japan). A professional photographer specialized in dental photography was consulted for setting the camera for intraoral shots. Thus, the camera was set with a focal length of 105 mm and an aperture of F/20 to F/22 and an exposure time of 1/125 seconds. The resolution of the images was 4,288 x 2,848 with 300 dpi. All images were pseudonymized and stored in an electronic database. Brightness, color or contrast of the images were not processed afterwards. However, the images of the buccal aspects and of the occlusal surfaces taken indirectly were mirrored to reflect the real positional relationship (Microsoft Photos 2020, Microsoft Corporation, Redmond, USA).

### Index test: Evaluation of digital dental photographs without and with panoramic radiographs

The pseudonymized photographs and panoramic radiographs were provided digitally to the clinical reference examiner and ten additional blinded raters for evaluation (index test). The blinded raters were seven dentists from the Department of Conservative Dentistry and three dental students from Heidelberg University. It was made sure to include assessors with different amount of clinical experience in oral diagnostics (Table 1).

**Table 1:** Examiners’ characteristics.

Variables	Clinical reference examiner (RE)	Blinded Raters: Experienced Dentists (n = 3)	Blinded Raters: Novice Dentists (n = 4)	Blinded Raters: Dental Students (n = 3)
**Clinical Experience**				
Mean ± SD (years)	7.08	10.75±1.26	2.96±0.48	1.58±0.00
Median (Q1 –Q3)	-	10.58 (9.58–12.08)	2.83 (2.58–3.33)	1.58 (1.58–1.58)

Abbreviations: RE = reference examiner, n = number of blinded raters, SD = standard deviation, Q = quartile, Min = Minimum, Max = Maximum

All examiners were trained in advance regarding the assessment and DMFT index reporting as proposed by the WHO using a separate case example. For the assessment of “Decayed”, all active carious lesions requiring treatment, e.g. primary or secondary caries, had to be considered. All the tooth surfaces had to be screened for lesions, including pit and fissures and smooth surfaces. It was noted that wisdom teeth were also included for this evalution. Assessments took place over a period of one week and without consultation between the blinded raters. In a first step, only the photographs were analysed (IODP). In a second step, the photographs were evaluated with additional consideration of the panoramic radiographs (IODP+PAN-X). The evaluation of the photographs and panoramic radiographs could be performed on the investigator’s own notebook or computer. The individual computer monitors and image viewer software used by the investigators are listed in S1 Table. For closer examination, individual processing of the photographs and the radiographs in terms of brightness and contrast as well as zooming in could be carried out independently. The documentation of the DMFT index and the number of implants was done on an evaluation sheet for IODP and IODP+PAN-X separately.

### Statistical analysis

Due to the exploratory nature of this pilot study on remote dental assessment in an elderly patient population, a sample size calculation has not been performed. In order to investigate the influence of the raters’ age on the agreement with clinical findings, ten blinded raters with differently amount of clinical experience have been chosen for remote assessment.

Patients’ demographic and clinical examination data were expressed descriptively. Continuous variables were expressed as mean ± standard deviation and categorical variables as absolute and relative frequencies.

In order to investigate the extent of agreement of the DMFT index between IODP with clinical diagnosis (primary endpoint), the clinical examination results were used as reference standard and hence, differences between the clinical diagnosis and the IODP and IODP+PAN-X assessment results were calculated and descriptively analysed using mean ± standard deviation and median (Q1-Q3).

Furthermore, to assess intra-rater agreement, pairwise Gwet’s AC1s of the three diagnostic methods “Clinical”, “IODP” and “IODP+PAN-X” were calculated separately for all examiners. In addition, overall pairwise Gwet’s AC1s were calculated using the mode of the ten blinded raters. Gwet’s AC1s were calculated because the widely used KAPPA statistics for testing the extent of agreement between raters yield unexpected low values in high agreement cases, which is present here [31, 32]. Gwet’s AC1 can be interpreted as follows: 0–0.2: slight agreement; 0.2–0.4: fair agreement; 0.4–0.6: moderate agreement; 0.6–0.8: substantial agreement; 0.8–1.0: almost perfect agreement [32, 33].

The secondary objective, whether the clinical experience of the raters influence the agreement of the assessment methods, was investigated using linear mixed effect models of the outcome “DMFT”. As dependent variable the difference between the clinical assessment and IODP or IODP+PAN-X of “DMFT” was calculated, e.g. a difference of zero indicates that the assessment methods led to the same DMFT values. As fixed effect (depend variable) the clinical experience of the raters (experienced/novices/students) was added to the model. Furthermore, random intercept effects for patient and examiner were added to the model to account for patient and examiner heterogeneity.

Statistical analyses were conducted using the statistic software R (version 4.0.2, R Core Team, Auckland, New Zealand) using the packages “lme4” and “lmerTest” for linear mixed effect models, “irrCAC” for calculation of Gwet’s AC1 and “ggplot” for data illustrations. Analyses were carried out at the Institute for Medical Biometry (IMBI) at Heidelberg University Hospital.

## Results

### Patients’ demographic and clinical examination data

The 50 study participants had a mean age of 70.98±7.60 years. Forty of them were female (80%). Clinical examination revealed a DMFT index of mean 22.10±3.75, “Decayed” of 0.18±0.52, “Missing” of 11.02±5.97, and Filled of “10.90±5.61”. The mean number of implants was 0.78±2.04.

### DMFT and implant assessment via intraoral digital photography

The IODP diagnosis by the clinical reference examiner and the ten blinded raters in relation to the DMFT index, “decayed”, “missing” and “filled” teeth as well as the number of implants are shown in Table 2 for each investigator and in the distinction between IODP alone and with PAN-X evaluation. The blinded raters’ value for DMFT was 21.54±3.40 by IODP and 22.12±3.45 by IODP+PAN-X compared to clinical diagnosis of 22.10±3.75. For “Decayed”, clinical examination revealed a value of 0.18±0.52 while the blinded raters assessed 0.45±0.46 (IODP) and 0.48±0.47 (IODP-PAN-X). Respectively, the means for “Missing” were 11.02±5.97 (clinical) compared to 10.66±5.78 (IODP) and 10.93±5.91 (IODP+PAN-X) whereas for “Filled”, they were 10.90±5.61 (clinical) versus 10.43±4.85 (IODP) and 10.71±5.11 (IODP+PAN-X). The assessment of number of implants revealed a mean of 0.78±2.04 by clinical examination and 0.58±1.43 (IODP) and 0.78±2.04 (IODP+PAN-X) by the blinded raters.

**Table 2:** DMFT and implant assessment via intraoral digital photography.

Examiners	Variables	DMFT		Decayed		Missing		Filled		Implants	
**Clinical Examination (RE)**	Mean ± SD	22.10±3.75		0.18±0.52		11.02±5.97		10.90±5.61		0.78±2.04	
	Median (Q1 –Q3)	22 (20–25)		0 (0–0)		10 (6–14)		12 (7–15)		0 (0–0)	
		**IODP**	**IODP+PAN-X**	**IODP**	**IODP+PAN-X**	**IODP**	**IODP+PAN-X**	**IODP**	**IODP+PAN-X**	**IODP**	**IODP+PAN-X**
**Reference Examiner (RE)**	Mean ± SD	21.54±3.62	22.26±3.55	0.16±0.42	0.16±0.42	10.98±6.07	11.04±5.98	10.40±5.60	11.06±5.60	0.76±2.05	0.78± 2.04
	Median (Q1 –Q3)	21 (20–24)	23 (20–25)	0 (0–0)	0 (0–0)	10 (6–14)	10 (6–14)	10.5 (6–15)	12 (6–15)	0 (0–0)	0 (0–0)
**Blinded Raters (all)**	Mean ± SD	21.54±3.40	22.12±3.45	0.45±0.46	0.48±0.47	10.66±5.78	10.93±5.91	10.43±4.85	10.71±5.11	0.58±1.43	0.78± 2.04
	Median (Q1 –Q3)	21.3 (19.6–23.4)	21.75 (19.8–24.3)	0.25 (0.10–0.80)	0.30 (0.10–0.80)	9.15 (6.3–13.6)	9.90 (6–14.1)	10.35 (6.3–14.2)	10.6 (6.2–14.8)	0 (0–0.3)	0 (0–0)
**Blinded Rater Exp_1**	Mean ± SD	21.80±3.48	22.20±3.55	0.66±0.80	0.70±0.84	10.66±5.79	10.78±5.83	10.48±5.31	10.72±5.30	0.70±1.85	0.78±2.04
	Median (Q1 –Q3)	21.5 (20–24)	22 (20–25)	0.50 (0–1.0)	0.50 (0–1.0)	10 (6–13)	10 (6–14)	10 (6–15)	11 (6–15)	0 (0–0)	0 (0–0)
**Blinded Rater Exp_2**	Mean ± SD	22.26±4.02	22.4±3.92	0.88±1.61	1.02±1.66	10.68±5.70	10.66±5.82	10.70±5.25	10.68±5.32	0.66±1.78	0.78±2.04
	Median (Q1 –Q3)	21 (20–25)	22 (20–24)	0 (0–1.0)	0 (0–1.0)	10 (6–14)	9.5 (5–14)	10.5 (7–15)	10.5 (7–14)	0 (0–0)	0 (0–0)
**Blinded Rater Exp_3**	Mean ± SD	21.36±4.08	21.76±3.99	0.18±0.48	0.18±0.48	9.92±5.79	10.94±6.00	11.26±5.83	10.64±5.53	0.16±0.65	0.74±2.04
	Median (Q1 –Q3)	21.5 (19–24)	22 (19–25)	0 (0–0)	0 (0–0)	9 (6–13)	9.5 (6–14)	11.5 (6–15)	11 (6–15)	0 (0–0)	0 (0–0)
**Blinded Rater Nov_1**	Mean ± SD	20.96±3.69	22.04±3.59	0.44±0.73	0.48±0.76	10.80±5.99	10.92±5.99	9.72±5.33	10.64±5.57	0.50±1.31	0.78±2.04
	Median (Q1 –Q3)	20 (19–23)	22 (19–24)	0 (0–1.0)	0 (0–1.0)	9 (6–14)	9 (6–15)	9.5 (6–14)	11 (6–15)	0 (0–0)	0 (0–0)
**Blinded Rater Nov_2**	Mean ± SD	21.60±4.28	21.96±3.97	0.72±1.03	0.74±1.05	11.10±5.96	11.02±6.02	9.78±5.11	10.20±5.42	1.00±2.02	0.78±2.04
	Median (Q1 –Q3)	21 (18–24)	22 (19–25)	0 (0–1.0)	0 (0–1.0)	9.5 (7–14)	10 (6–14)	10.5 (6–14)	10.5 (6–15)	0 (0–1)	0 (0–0)
**Blinded Rater Nov_3**	Mean ± SD	20.96±3.48	21.58±3.70	0.28±0.70	0.32±0.77	10.62±6.00	10.86±5.96	10.06±5.43	10.40±5.32	0.64±1.74	0.78±2.04
	Median (Q1 –Q3)	21 (19–23)	21 (19–24)	0 (0–0)	0 (0–0)	9 (7–13)	9.5 (6–14)	10 (6–14)	10 (6–14)	0 (0–0)	0 (0–0)
**Blinded Rater Nov_4**	Mean ± SD	22.38±3.69	22.88±3.71	0.76±1.04	0.76±1.04	10.80±5.95	10.98±5.94	10.82±5.04	11.14±4.94	0.56±1.64	0.78±2.04
	Median (Q1 –Q3)	22 (20–24)	23 (21–25)	0 (0–1.0)	0 (0–1.0)	9.5 (6–14)	9.5 (6–14)	10 (7–15)	12 (7–14)	0 (0–0)	0 (0–0)
**Blinded Rater Stud_1**	Mean ± SD	21.66±3.32	22.34±3.42	0.28±0.61	0.28±0.61	10.42±5.58	11.06±5.94	10.96±4.97	11.00±5.29	0.32±1.13	0.78±2.04
	Median (Q1 –Q3)	21.5 (19–24)	22 (19–25)	0 (0–0)	0 (0–0)	10 (6–13)	9.5 (6–14)	11.5 (7–14)	11 (6–15)	0 (0–0)	0 (0–0)
**Blinded Rater Stud_2**	Mean ± SD	21.68±4.03	22.32±3.87	0.16±0.37	0.16±0.37	10.68±5.92	11.06±5.97	10.84±4.78	11.10±5.00	0.52±1.52	0.78±2.04
	Median (Q1 –Q3)	21.5 (20–24)	22 (20–25)	0 (0–0)	0 (0–0)	9 (6–14)	10.5 (6–14)	11 (7–14)	10.5 (7–16)	0 (0–0)	0 (0–0)
**Blinded Rater Stud_3**	Mean ± SD	20.72±3.82	21.72±3.74	0.12±0.33	0.12±0.33	10.94±6.00	11.02±5.97	9.66±5.17	10.58±5.55	0.72±2.03	0.78±2.04
	Median (Q1 –Q3)	20.5 (18–23)	22 (20–24)	0 (0–0)	0 (0–0)	9 (6–14)	10 (6–14)	9 (6–14)	10 (6–15)	0 (0–0)	0 (0–0)

Abbreviations: SD = standard deviation, Q = quartile, IODP = intraoral digital photography, PAN-X = panoramic radiograph), RE = reference examiner, Exp = Experienced Dentist, Nov = Novice Dentist, Stud = Dental Student

Agreement between clinical examination and IODP/IODP+PAN-X diagnostics on the individual patient level is shown with pairwise Gwet’s AC1 for the clinical reference examiner and for each of the blinded raters (Table 3).

**Table 3:** Agreement of IODP/IODP+PAN-X assessment and clinical diagnosis.

		DMFT	Decayed	Missing	Filled	Implants
**Clinical Examination vs. Imaging Assessment (RE)** [Gwet AC1 (95% CI)]	IODP	0.361 (0.212, 0.511)	0.826 (0.707, 0.945)	0.895 (0.805, 0.985)	0.394 (0.246, 0.542)	0.979 (0.935, 1.000)
	IODP + PAN-X	0.576 (0.427, 0.725)	0.826 (0.707, 0.945)	0.979 (0.937, 1.000)	0.540 (0.391, 0.689)	1.000 (1.000, 1.000)
**Clinical Examination vs. Imaging Assessment (BR_all)** [Gwet AC1 (95% CI)]	IODP	0.364 (0.214, 0.513)	0.848 (0.737, 0.96)	0.812 (0.696, 0.927)	0.290 (0.15, 0.43)	0.895 (0.802, 0.987)
	IODP + PAN-X	0.364 (0.214, 0.513)	0.871 (0.767, 0.974)	0.958 (0.899, 1.000)	0.331 (0.187, 0.475)	1.000 (1.000, 1.000)
**Clinical Examination vs. Imaging Assessment (BR_Exp_1)** [Gwet AC1 (95% CI)]	IODP	0.300 (0.156, 0.444)	0.478 (0.296, 0.660)	0.728 (0.596, 0.860)	0.228 (0.097, 0.360)	0.914 (0.829, 0.999)
	IODP + PAN-X	0.258 (0.118, 0.397)	0.452 (0.266, 0.638)	0.853 (0.749, 0.958)	0.309 (0.167, 0.451)	1.000 (1.000, 1.000)
**Clinical Examination vs. Imaging Assessment (BR_Exp_2)** [Gwet AC1 (95% CI)]	IODP	0.091 (-0.014, 0.197)	0.574 (0.419, 0.729)	0.686 (0.548, 0.824)	0.083 (-0.015, 0.180)	0.958 (0.897, 1.000)
	IODP + PAN-X	0.113 (0.001, 0.224)	0.529 (0.371, 0.687)	0.749 (0.621, 0.877)	0.081 (-0.017, 0.178)	1.000 (1.000, 1.000)
**Clinical Examination vs. Imaging Assessment (BR_Exp_3)** [Gwet AC1 (95% CI)]	IODP	0.239 (0.103, 0.375)	0.782 (0.649, 0.915)	0.541 (0.392, 0.689)	0.248 (0.113, 0.382)	0.812 (0.693, 0.931)
	IODP + PAN-X	0.385 (0.234, 0.535)	0.782 (0.649, 0.915)	0.874 (0.777, 0.972)	0.373 (0.226, 0.520)	0.979 (0.935, 1.000)
**Clinical Examination vs. Imaging Assessment (BR_Nov_1)** [Gwet AC1 (95% CI)]	IODP	0.151 (0.029, 0.273)	0.611 (0.442, 0.780)	0.728 (0.596, 0.860)	0.081 (-0.017, 0.178)	0.894 (0.801, 0.987)
	IODP + PAN-X	0.172 (0.046, 0.299)	0.609 (0.439, 0.780)	0.791 (0.670, 0.911)	0.228 (0.097, 0.360)	1.000 (1.000, 1.000)
**Clinical Examination vs. Imaging Assessment (BR_ Nov_2)** [Gwet AC1 (95% CI)]	IODP	0.302 (0.158, 0.446)	0.482 (0.307, 0.657)	0.728 (0.596, 0.860)	0.081 (-0.017, 0.178)	0.766 (0.635, 0.896)
	IODP + PAN-X	0.260 (0.121, 0.399)	0.482 (0.307, 0.657)	0.937 (0.866, 1.000)	0.227 (0.095, 0.358)	1.000 (1.000, 1.000)
**Clinical Examination vs. Imaging Assessment (BR_Nov_3)** [Gwet AC1 (95% CI)]	IODP	0.172 (0.046, 0.299)	0.846 (0.732, 0.960)	0.582 (0.435, 0.729)	0.164 (0.044, 0.284)	0.808 (0.687, 0.929)
	IODP + PAN-X	0.364 (0.214, 0.513)	0.800 (0.672, 0.928)	0.833 (0.722, 0.943)	0.372 (0.225, 0.519)	1.000 (1.000, 1.000)
**Clinical Examination vs. Imaging Assessment (BR_Nov_4)** [Gwet AC1 (95% CI)]	IODP	0.220 (0.087, 0.353)	0.505 (0.322, 0.687)	0.644 (0.502, 0.787)	0.077 (-0.021, 0.174)	0.853 (0.746, 0.960)
	IODP + PAN-X	0.326 (0.180, 0.471)	0.505 (0.322, 0.687)	0.833 (0.722, 0.943)	0.163 (0.042, 0.283)	1.000 (1.000, 1.000)
**Clinical Examination vs. Imaging Assessment (BR_Stud_1)** [Gwet AC1 (95% CI)]	IODP	0.109 (-0.003, 0.221)	0.667 (0.510, 0.825)	0.561 (0.413, 0.709)	0.079 (-0.019, 0.176)	0.873 (0.771, 0.974)
	IODP + PAN-X	0.173 (0.046, 0.299)	0.667 (0.510, 0.825)	0.874 (0.777, 0.972)	0.060 (-0.030, 0.150)	1.000 (1.000, 1.000)
**Clinical Examination vs. Imaging Assessment (BR_Stud_2)** [Gwet AC1 (95% CI)]	IODP	0.241 (0.105, 0.378)	0.793 (0.658, 0.929)	0.727 (0.595, 0.859)	0.225 (0.094, 0.357)	0.852 (0.745, 0.960)
	IODP + PAN-X	0.326 (0.180, 0.471)	0.793 (0.658, 0.929)	0.874 (0.777, 0.972)	0.267 (0.130, 0.405)	1.000 (1.000, 1.000)
**Clinical Examination vs. Imaging Assessment (BR_ Stud_3)** [Gwet AC1 (95% CI)]	IODP	0.257 (0.118, 0.397)	0.842 (0.723, 0.961)	0.937 (0.866, 1.000)	0.288 (0.148, 0.428)	0.936 (0.862, 1.000)
	IODP + PAN-X	0.383 (0.232, 0.533)	0.842 (0.723, 0.961)	1.000 (1.000, 1.000)	0.415 (0.266, 0.564)	1.000 (1.000, 1.000)

Abbreviations: RE = reference examiner, BR = blinded raters, IODP = intraoral digital photography, PAN-X = panoramic radiograph, CI = confidence interval, Exp = Experienced Dentist, Nov = Novice Dentist, Stud = Dental Student; lighter grey shades = higher agreement; darker grey shades = lower agreement

When measuring the agreement of the blinded raters’ assessment of "Decayed", "Missing" and "Implants" with the clinical reference findings, overall Gwet’s AC1 using the mode of the blinded raters ranged from 0.81 to 0.89 (IODP) and 0.87 to 1.00 (IODP+PAN-X), while for "Filled" and "DMFT" they were 0.29 and 0.36 (IODP) as well as 0.33 and 0.36 (IODP+PAN-X), respectively. For comparative evaluation of the reference examiner’s assessment, Gwet’s AC1s for "Decayed", "Missing" and "Implants" ranged from 0.83 to 0.98 (IODP) and 0.83 to 1.00 (IODP+PAN-X). For "Filled" and "DMFT", Gwet’s AC1s ranged from 0.36 to 0.39 (IODP) and 0.54 to 0.58 (IODP+PAN-X), respectively.

**Fig 2:**
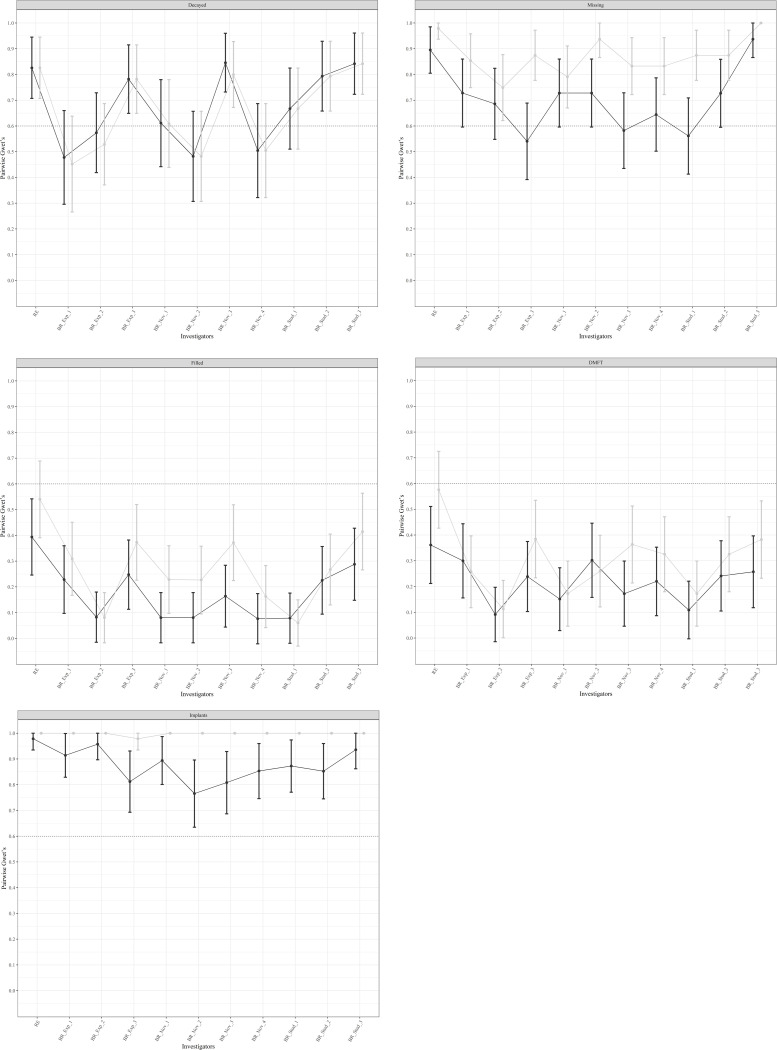
Gwet AC1-Plots on the agreement of clinical reference standard with remote assessment. (A) Decayed, (B) Missing, (C) Filled, (D) DMFT, (E) number of implants, Abbreviations: IODP = intraoral digital photography, PAN-X = panoramic radiograph, RE = reference examiner, BR = blinded raters, Exp = Experienced Dentist, Nov = Novice Dentist, Stud = Dental Student.

Fig 2 highlights the benefit of the additional evaluation of the panoramic radiographs to IODP for almost every comparison and investigator.

### Investigator’s clinical experience

When comparing the differences between clinical results as reference standard and the IODP/IODP+PAN-X asssessment by the blinded raters, the linear mixed effect models for “DMFT” show no statistically significant differences between experienced dentists, novice dentists and dental students (S2 Table).

## Discussion

In this elderly study population with advanced restorative and periodontal status, assessment of the DMFT index as well as the number of implants by means of digital dental photographs, showed miscellaneous agreement compared with clinical diagnosis. Comparisons of the parameters "Decayed", "Missing" and "Implants" show almost perfect agreement by IODP without and with the aid of the panoramic radiographs—this applies to the distinction between the clinical reference examiner alone and between the reference examiner and the blinded raters. For "Filled" and accordingly "DMFT", there is moderate agreement between the clinical examination and the IODP/IODP+PAN-X diagnosis by the reference examiner supported by the radiographic diagnostics, whereas without radiographic evaluation there is fair agreement. With respect to the IODP/IODP+PAN-X evaluation by the blinded raters, only fair agreement on these two parameters is found, regardless of whether the photodiagnostic was performed without or with radiographic evaluation.

In the overall patient cohort, mean and median values in all the examined diagnostic parameters were very similar between clinical diagnosis and the assessment of the blinded raters, while the agreement between IODP/IODP+PAN-X-diagnosis on an individual patient level was lower for “Filled” and “DMFT” compared to “Decayed”, “Missing” and “Implants”. The additional assessment of the panoramic radiographs led to higher agreement in most parameters and in almost all investigators.

While the clinical experience of the investigators showed no influence on the agreement, slightly higher agreement of the clinical examiner compared to the blinded raters was found in each parameter. This may be due to several reasons. Given the high number of photographs and patients, the effect of unblinding is unlikely. It is more plausible that the individual decision for the need for treatment may also play a role, for example in caries diagnostics. It has been shown that regardless of the clinical experience, the subjective assessment can be more or less conservative [34]. This also illustrates the assumption that–even with strict criteria for defining caries or insufficient restorations–for multi-center epidemiological studies, a bias in the results can be reduced if one designated examiner alone makes the distinct decisions. For the rather objective parameters “Missing”, “Filled” and “Implants”, this should play a minor role.

The lower agreement for "Filled" and "DMFT" despite similar mean values in the descriptive analysis can be explained by the greater variance, as can be seen from the higher standard deviations and the first and third quartiles. One reason for the somewhat higher scatter of the findings for "Filled" could be the lack of tactile inspection of the tooth surfaces for restoration margins and the reduced visualization on intraoral photographs of proximal and lingual areas. The use of bitewing instead of panoramic radiographs could result in a more precise assessment of “filled” teeth. Also, the additional analysis of lateral photographs taken with an open bite could help detecting restorations or carious lesions in the coronal or incisal aspects of the teeth. The difficulty in detecting esthetic restorations should also be highlighted. Signori et al. investigated photodiagnostics with regard to the assessment of repair and replacement needs or failure of restorations compared to clinical diagnostics and showed higher agreement in the posterior region compared to the anterior region with more esthetic restorations [35].

Other studies, however, emphasize that the assessment of restorations or tooth defects via intraoral photographs could be more accurate compared to clinical examination, taking advantage of digital post-processing and magnification of the photographs [28, 36].

Compared to other studies that investigated oral diagnostics by means of dental photographs, the subjects of the present study were older and had a higher restorative status [18, 19, 21–23, 25, 28]. This increases the external validity of the study and the generalizability of the findings to other age groups. For example, the current Fifth German Oral Health Study (DMS V) showed a DMFT of 17.7, "Decayed" of 0.5, "Missing" of 11.1 and "Filled" of 6.1 in seniors aged 65–74 years, respectively [37]. Our cohort showed similar values for "Missing" (11.02±5.97) and for "Decayed" (0.18±0.52). However, "Filled" showed higher values of 10.90±5.61 and the overall DMFT index was 22.10±3.75. In contrast to DMS V, wisdom teeth were included in the present study. The mean number of implants in this cohort was 0.78±2.04, whereas in the DMS V 0.22 implants were determined in the corresponding age group [37]. It should be noted that the patients in the present study were recruited in a university department specialized in conservative dentistry.

### Strengths and limitations

In addition to the elderly patient population, another strength of this study is the standardized clinical examination and photographs taken by one experienced investigator. Further, ten blinded examiners with varying levels of clinical experience participated in data acquisition. Thus, a comparison between blinded raters and the clinical reference examiner as well as of the reference examiner alone could be investigated. The study design also allows to draw conclusions on the diagnostic performance of intraoral digital photography alone and in combination with the evaluation of panoramic radiographs.

The blinded examiners were able to rate the digital images on their own monitors. The photographs could be adjusted in brightness and contrast or zoomed in according to individual needs. On the one hand, this represents a limitation of internal validity; on the other hand, it increases the generalizability of the findings for large-scale epidemiological study projects in which different terminal devices are also used for data collection. An in vitro study on caries diagnosis using intraoral photographs also demonstrated no significant differences in the results when distinguishing between standardized and non-standardized viewing conditions [38].

Since this study cohort demonstrates a low number of teeth with caries or restorations in need of renewal, a valid statement on the detection of “Decayed” cannot be provided.

## Conclusions

For large-scale epidemiological studies, this method of diagnostics by intraoral digital photography in an elderly patient population with advanced restorative status might be suitable, keeping in mind some limitations of the present study. Further studies including patients with a higher incidence of caries or restorations in need of replacement would be necessary. A further investigation of different additional radiological evaluation tools as bitewing radiographs would also be helpful in order to increase the accuracy of “filled” teeth.

## Supporting information

S1 Table Computer monitor and image viewer software used for image assessment. (PDF)

S2 Table Linear mixed effect model for “DMFT” (n = 500). (PDF)

S1 Checklist (DOCX)

S2 Checklist STROBE statement—Checklist of items that should be included in reports of cross-sectional studies. (DOC)
