# Supplementary material for: Remote assessment of DMFT and number of implants with intraoral digital photography in an elderly patient population – a comparative study
Source: PLoS One. 2022 May 17;17(5):e0268360. doi: 10.1371/journal.pone.0268360 (PMC9113588; doi:10.1371/journal.pone.0268360)
Supplement: S1 Table — (PDF) [file pone.0268360.s001.pdf]

S1 Table. Computer monitor and image viewer software used for image assessment

Investigator	Computer monitor	Image viewer software
Reference Examiner	LG 23MB35PY-W<br>(LG Electronics, Seoul, South Korea)	Microsoft Photos 2020<br>(Microsoft Corporation, Redmond, USA)
Blinded Rater Exp_1	Microsoft Surface Pro 4<br>(Microsoft Corporation, Redmond, USA)	Microsoft Photos 2020<br>(Microsoft Corporation, Redmond, USA)
Blinded Rater Exp_2	EIZO FlexScan EV2450<br>(EIZO Corporation, Hakusan, Japan)	Microsoft Photos 2020<br>(Microsoft Corporation, Redmond, USA)
Blinded Rater Exp_3	LG 23MB35PY-W<br>(LG Electronics, Seoul, South Korea)	Microsoft Photos 2020<br>(Microsoft Corporation, Redmond, USA)
Blinded Rater Nov_1	LG 23MB35PY-W<br>(LG Electronics, Seoul, South Korea)	Microsoft Photos 2020<br>(Microsoft Corporation, Redmond, USA)
Blinded Rater Nov_2	EIZO FlexScan EV2450<br>(EIZO Corporation, Hakusan, Japan)	Microsoft Photos 2020<br>(Microsoft Corporation, Redmond, USA)
Blinded Rater Nov_3	LG IPS231P-BN<br>(LG Electronics, Seoul, South Korea)	Microsoft Photos 2020<br>(Microsoft Corporation, Redmond, USA)
Blinded Rater Nov_4	MacBookPro11,3 (2014)<br>(Apple Inc., Cupertino, USA)	Preview 2020<br>(Apple Inc., Cupertino, USA)
Blinded Rater Stud_1	Lenovo Thinkpad T460<br>(LenovoLenovo Group Limited, Hongkong, China	Microsoft Photos 2020<br>(Microsoft Corporation, Redmond, USA)
Blinded Rater Stud_2	MacBookAir9,1<br>(Apple Inc., Cupertino, USA)	Preview 2020<br>(Apple Inc., Cupertino, USA)
Blinded Rater Stud_3	MacBookPro9,2<br>(Apple Inc., Cupertino, USA)	Preview 2020<br>(Apple Inc., Cupertino, USA)

Abbreviations: Exp = Experienced Dentist, Nov = Novice Dentist, Stud = Dental Student
