# Supplementary material for: Remote assessment of DMFT and number of implants with intraoral digital photography in an elderly patient population – a comparative study
Source: PLoS One. 2022 May 17;17(5):e0268360. doi: 10.1371/journal.pone.0268360 (PMC9113588; doi:10.1371/journal.pone.0268360)
Supplement: S2 Table — (PDF) [file pone.0268360.s002.pdf]

S2 Table. Linear mixed effect model for “DMFT” (n=500)

	Estimate	Lower 95% CI	Upper 95% CI	p-Value
		Clinical diagnosis vs. IODP		
Intercept	0.293	-0.420	1.007	0.460
Experienced Dentists	0			
Novice Dentists	0.332	-0.500	1.163	0.480
Dental Students	0.453	-0.436	1.343	0.372
		Clinical diagnosis vs. IODP+PAN-X		
Intercept	-0.007	-0.579	0.566	0.983
Experienced Dentists	0			
Novice Dentists	-0.008	-0.638	0.622	0.981
Dental Students	-0.020	-0.693	0.653	0.957

Abbreviations: CI = confidence interval, IODP = intraoral digital photography, PAN-X = panoramic radiograph
